# Correlation analysis of preoperative renal insufficiency with major complications in patients who received a radical cystectomy and pelvic lymph node dissection: results of a retrospective observational analysis from a single center

**DOI:** 10.3389/fonc.2024.1453346

**Published:** 2024-11-22

**Authors:** Haixin Wang, Haiwen Huang, Han Hao, Zhijun Xi

**Affiliations:** ^1^ Department of Urology, Peking University First Hospital, Beijing, China; ^2^ Institute of Urology, Peking University, National Urological Cancer Center, Beijing, China; ^3^ National Research Center for Genitourinary Oncology, Beijing, China; ^4^ Department of Urology, Yankuang New Journey General Hospital, Zoucheng, Shandong, China; ^5^ Department of Urology, Tsinghua University Affiliated Beijing Tsinghua Changgung Hospital, Beijing, China

**Keywords:** radical cystectomy, renal function, renal insufficiency, complications, Clavien-Dindo

## Abstract

**Objective:**

The aim of this study was to explore the factors affecting the major complications and the impacts of preoperative renal function on the incidence of complications in radical cystectomy procedures.

**Methods:**

A retrospective review of 705 patients who received radical cystectomy between 2006 and 2021 was conducted. The 90-day complications of patients after a radical cystectomy were reported and the Clavien–Dindo classification (CDC) was used for grading complications. The clinical characteristics and preoperative outcomes were compared among patients with different preoperative renal functions. A logistic regression analysis of all patients was used to identify the risk factors associated with the major complications. Spearman’s correlation analysis was used to examine the relationship between the classification of renal insufficiency and the CDC. In order to reduce the selection bias, one-to-one propensity score matching was performed, and the comparison of complications after matching was carried out for the sensitivity analysis.

**Results:**

Within 90 days post-surgery, 71% of patients experienced complications, with 4.8% of them being major. Patients with preoperative renal insufficiency had a higher CDC and had a higher rate of major complications (16.7% vs 3.7%, *p* < 0.001). There was a linear relationship between preoperative serum creatinine and complications. Spearman’s correlation analysis showed a slightly positive correlation between the classification of renal insufficiency and the CDC (r=0.094, *p* = 0.013). Preoperative renal insufficiency was a risk factor for major complications (OR = 6.805 [95%CI: 2.706-17.112]; *p* < 0.001). After matching, the patients in the preoperative renal insufficiency group had a higher CDC and a higher incidence of major complications (16.9% vs 1.7%, p = 0.004).

**Conclusions:**

In our cohort, patients with preoperative renal insufficiency exhibited a higher incidence of complications following a radical cystectomy, and renal insufficiency was a significant risk factor for major complications.

## 1 Introduction

Bladder cancer is the sixth most common genitourinary malignant disease in the United States with 81,180 estimated new cases and 17,100 estimated deaths in 2022 (1). A radical cystectomy with pelvic lymph node dissection (PLND) is the gold standard for muscle-invasive bladder cancer and recurrent high-grade non-muscle-invasive bladder cancer (2). However, its morbidity from complications was as high as 31.5%-64% because of the surgical trauma (3–11). Minimally invasive surgery can achieve comparable oncological control while reducing the incidence of complications and shortening hospital stays (12–15). Notably, studies have demonstrated that the adoption of a completely minimally invasive approach in the surgical treatment of bladder cancer has led to an overall reduction in transfusion rates of 50% (14, 16). It is still a highly comorbid operation, with a risk of major complications in at least one-third of patients in contemporary practice (4).

Previous studies have identified several factors, including urinary diversion (3, 11), the American Society of Anesthesiologists (ASA) score (6), the Charlson comorbidity index, and preoperative coagulation functions (10), that influence complications post-radical cystectomy. Interestingly, the presence of preoperative renal insufficiency was associated with major complications in elderly patients in our previous study (17, 18). However, it is not clear whether preoperative renal insufficiency increases the incidence of complications after a radical cystectomy.

The aim of this study was to compare the incidence of complications among patients with different preoperative renal functions and explore the factors affecting major complications in radical cystectomy procedures.

## 2 Methods

### 2.1 Patient population

**Figure 1:**
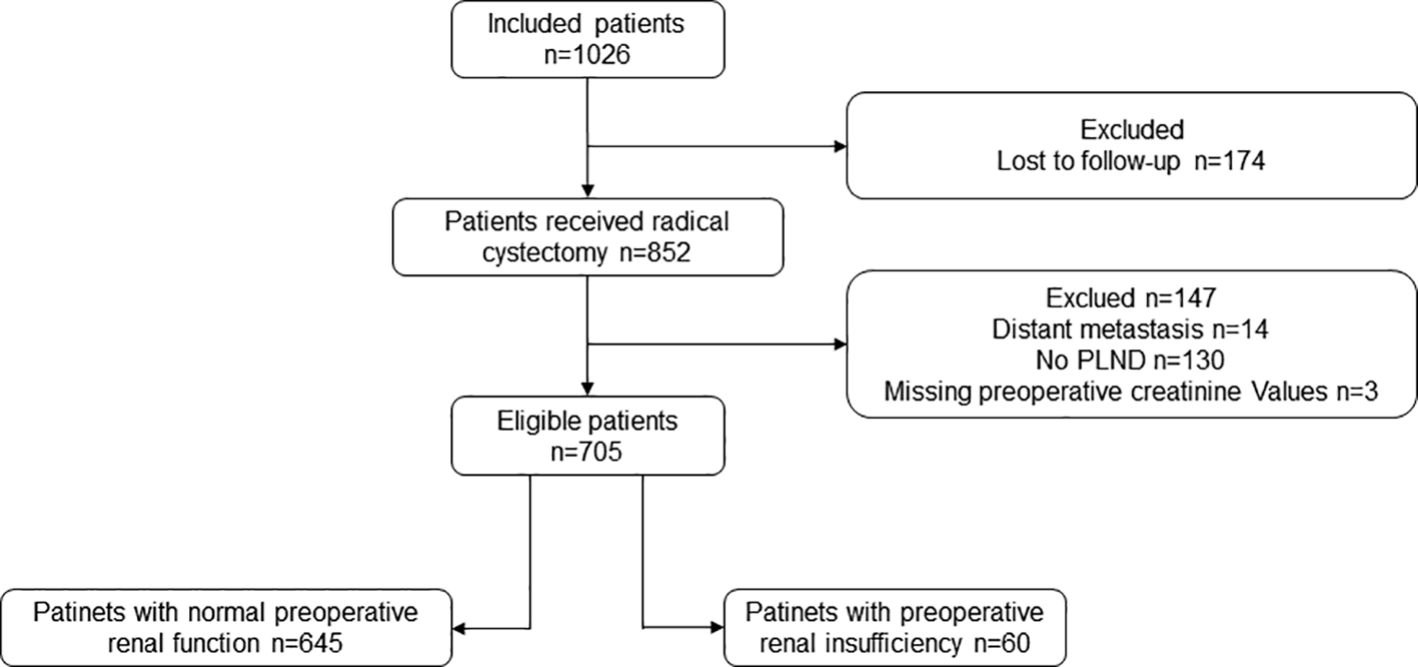
Flowchart of all eligible patients. PLND, pelvic lymph node dissection.

A retrospective analysis of morbidity data from chart review was performed in this study. We defined the following inclusion criteria (1): patients who underwent a radical cystectomy from January 2006 to April 2017 at Peking University First Hospital; and (2) postoperative pathology indicated urothelial carcinoma of the bladder. The exclusion criteria were (1) distant metastasis (n=14); (2) PLND not performed (n=130); and (3) lack of preoperative creatinine data (n=3). Finally, 705 patients who had received a radical cystectomy and PLND were enrolled (**Figure 1**). Among them, 60 (8.5%) patients had preoperative renal insufficiency (serum creatinine > 133umol/L) (19) and 645 (91.5%) had a normal preoperative renal function. Imaging and pathological examinations were performed for all patients before the operation. The indications for a radical cystectomy included a T2-4aN_x_M0 tumor, high-risk and recurrent non-muscleinvasive bladder cancer (NMIBC), and BCG-resistant Tis, as well as extensive papillary disease that could not be controlled with a transurethral resection of the bladder tumor and intravesical therapy alone. Patients with a cT2-4aN0-xM0 tumor were recommended for neoadjuvant chemotherapy, whereas adjuvant chemotherapy was recommended for patients with pT3/4 or pN+ disease if no neoadjuvant chemotherapy had been given. The final treatment plans were determined according to the patient’s tolerance and preference. This study was approved by the clinical research ethics committee of Peking University First Hospital, Beijing, China.

### 2.2 Outcomes measures

#### 2.2.1 Clinical and pathological data

The preoperative serum creatinine (sCr) was collected for all patients, and sCr ≥ 133 umol/L was defined as preoperative renal insufficiency. According to the preoperative renal function, the patients were categorized into two groups: the normal renal function group and the preoperative renal insufficiency group. Clinical characteristics including age, sex, operation method (open radical cystectomy or laparoscopic radical cystectomy), the ASA score (20), time of operation, postoperative stay, estimated blood loss (EBL), body mass index (BMI), and urinary diversion type were collected in this study. Histological type, surgical margin status, lymph node yield, and number of positive lymph nodes were collected in pathological data. Pathological T stage and pathological nodal stage were classified by using the TNM staging system of bladder cancer of the American Joint Committee on Cancer Staging Manual 8th edition. The K/DOQI (clinical practice guidelines for chronic kidney disease) grades specific disease and conditions into four grades: grade 1—compensatory stage of renal insufficiency (177umol/L ≥ sCr > 133umol/L); grade 2—decompensatory stage of renal insufficiency (443umol/L ≥ sCr > 177umol/L); grade 3— renal failure stage (707umol/L ≥ sCr > 443umol/L); grade 4— uremia stage (sCr > 707umol/L) (19).

#### 2.2.2 Surgical approach

The surgical approach was conducted as previously described in the literature (13). The choice of operative method—open radical cystectomy (ORC) or laparoscopic radical cystectomy (LRC)—was determined by the patient’s condition and the surgeon’s preference. The entire procedure involved an en-bloc resection of the bladder, a bilateral PLND, and extracorporeal urinary diversion. PLND encompassed the removal of internal iliac, presacral, obturator, and external iliac lymph nodes, and urinary diversion included options such as a ureterocutaneostomy, ileal conduit, or orthotopic neobladder. The selection of the urinary diversion type was based on the tumor stage and the patient’s physical condition and was decided upon after thorough discussions between the medical team and the patient. However, the option of an orthotopic neobladder was excluded in cases where there was suspicion of urethral invasion.

#### 2.2.3 Assessment of complications

The collection and grading of complications were conducted by two clinical doctors. Definitions of complications after radical cystectomy were from the catalog of studies by Vetterlein et al. (21), which employed the Common Terminology Criteria for Adverse Events (CTCAE) v5.0. The 90-day complications after a radical cystectomy were collected from digitalized charts and outpatient information was included. Each complication was graded using the Clavien–Dindo classification (CDC) (22). A major complication was characterized as CDC III-V (10).

### 2.3 Statistical analysis

Statistical analysis was performed using Statistical Package for the Social Sciences (SPSS) version 25.0 software (IBM Corporation, Armonk, NY, USA). All *p*-values were two-sided and *p* < 0.05 was considered to be statistically significant. For the continuous variables, the Kolmogorov–Smirnov test was used to identify the normality. Continuous variables were presented as means ± standard deviation for normal distributions, or as medians and interquartile ranges (IQR) for skewed distributions. The independent t-test was used to compare the variables following a normal distribution, and variables following a non-normal distribution were analyzed using the Mann–Whitney U test. The *x* ^2^ test or Fisher’s exact test was used to compare the disordered categorical variables, while the ordered categorical variables were analyzed by using the Mann–Whitney U test. A logistic regression analysis of all patients was used to identify the risk factors associated with the major complications. Spearman’s correlation analysis was used to examine the relationship between the classification of renal insufficiency and the CDC. Concurrently, for the sensitivity analysis, a one-to-one matching was conducted using propensity scores. In estimating the propensity scores, a logistic regression model was employed, incorporating the following factors: age, ASA score, type of urinary diversion, the pathological T stage, and the pathological nodal stage. A caliper width of 0.02 was applied for the propensity score matching (PSM). Post-matching, the CDC of the patients and the incidence of major complications were compared.

## 3 Results

### 3.1 Patient characteristics

There were 705 patients enrolled in the present study. Among them, 645 (91.5%) patients had a normal preoperative renal function and 60 (8.5%) had an elevated sCr level higher than the normal value. There were 501 (71%) patients who experienced at least one complication within 90 days of the surgery and 34 (4.8%) patients had major complications.

The perioperative and pathological outcomes are presented in **Table 1**. Compared with the normal renal function group, the patients with preoperative renal insufficiency had higher ASA scores (*p* < 0.001), higher clinical T stages (*p* < 0.001), higher pathological T stages (*p* < 0.001), and higher pathological nodal stages (25% vs 14.3%, *p* = 0.027). Furthermore, more patients with preoperative renal insufficiency underwent a ureterocutaneostomy (43.3% vs 25.7%, *p* = 0.014) and had a higher rate of positive margins (6.7% vs 1.6%, *p* = 0.026) after a radical cystectomy.

**Table 1:** The clinicopathologic characteristics of all patients.

	Normal renal function(n =645)	Preoperative renal insufficiency(n =60 )	*p*
Male	553 (85.7%)	54 (90.0%)	0.361
Age	67 (58-73)	69 (60-76)	0.067
ORC	451 (69.9%)	41 (68.3%)	0.798
LRC	194 (30.1%)	19 (31.7%)	
BMI (kg/m^2^)	23.9 (21.8-26.3)	24.3 (22.2-26.0)	0.443
ASA score			<0.001
≤2	552 (85.6%)	398(63.3%)	
>2	93(14.4%)	22(36.7)	
Type of urinary diversion			0.014
Ureterocutaneostomy	166 (25.7%)	26 (43.3%)	
Ileal conduit	447 (69.3%)	33 (55.0%)	
Orthotopic neobladder	32 (5%)	1 (1.7%)	
Time of operation (min)	302 (230-374)	269 (209-354)	0.07
EBL (ml)	500(200-1000)	600 (200-1000)	0.785
Clavien-Dindo class			0.001
0	190 (29.5%)	14(23.3%)	
1	7(1.1%)	0	
2	424 (65.7%)	36 (60.0%)	
3	17 (2.6%)	4 (6.7%)	
4	6 (0.9%)	5 (8.3%)	
5	1 (0.2%)	1 (1.7%)	
Major complication	24 (3.7%)	10 (16.7%)	<0.001
Postoperative stay (days)	10 (8-14)	11 (8-15)	0.938
Clinical T stage			<0.001
Ta and Tis and T1	194 (30.1%)	5 (8.3%)	
T2	226(35.0%)	16 (26.7%)	
T3	142 (22.0%)	20 (33.3%)	
T4	83 (12.9%)	19 (31.7%)	
Pathological T stage			<0.001
Ta and Tis and T1	189 (29.3%)	5 (8.3%)	
T2	204 (31.6%)	15 (25.0%)	
T3	195(30.2%)	30 (50.0%)	
T4	57(8.8%)	10 (16.7%)	
Pathologic nodal stage			0.027
N0	553(85.7%)	45(75%)	
N+	92(14.3%)	15(25%)	
Pathologic grade			0.613
Low grade	67 (10.4%)	5 (8.3%)	
High grade	577 (89.6%)	55 (91.7%)	
Negative margin	635 (98.4%)	56(93.3%)	0.026
Positive margin	10 (1.6%)	4 (6.7%)	
Chemotherapy	126 (19.5%)	9 (15.0%)	0.393
No chemotherapy	519 (80.5%)	51 (85.0%)	

ORC, open radical cystectomy; LRC , laparoscopic radical cystectomy; BMI, Body Mass Index; EBL, estimated blood loss; ASA, American Society of Anesthesiologists.

### 3.2 Postoperative complications

A total of 501 (71.16%) patients suffered from complications after the operation. Among them, 34 patients had more than one major complication. Compared with the group with normal renal function, the patients with preoperative renal insufficiency also had higher CDCs and had higher rate of major complications (16.7% vs 3.7%, *p* < 0.001). Spearman’s correlation analysis showed a slightly positive correlation between the classification of renal insufficiency and the CDC (r=0.094, *p* = 0.013).

To further identify the risk of the postoperative major complications, multivariate logistic regression modeling was performed. We found preoperative renal insufficiency (OR = 6.805 [95%CI: 2.706-17.112]; *p* < 0.001) was associated with a higher risk of major complications (**Table 2**).

**Table 2:** Logistic regression analysis of variables associated with major complication.

	Odds Ratio	95% CI	*p*
ASA score			
≤2	-	referent	-
>2	2.482	1.050-5.865	0.038
Pathological T stage			0.031
Ta and Tis and T1	-	referent	-
T2	0.269	0.102-0.710	0.008
T3	0.345	0.135-0.884	0.027
T4	0.141	0.007-2.927	0.206
Pathologic nodal stage			
N0	-	referent	-
N+	0.717	0.077-5.837	0.717
Preoperative renal sufficien			
No	-	referent	-
Yes	6.805	2.706-17.112	<0.001
Type of urinary diversion			0.705
ureterocutaneostomy	-	referent	-
Ileal conduit	0.818	0.325-2.060	0.67
Orthotopic neobladder	1.47	0.213-10.155	0.696
Age	1.003	1.000-1.007	0.391
Time of operation (mins)	1.004	1.000-1.007	0.07

Ultimately, we conducted a comparison of the types of major complications between patients with normal preoperative renal function and those with preoperative renal insufficiency (**Table 3**). Most major complications occurred within 30 days postoperatively, with only two patients in the normal preoperative renal function group and one patient in the preoperative renal insufficiency group experiencing major complications beyond 30 days. Our findings revealed that among the patients with preoperative renal insufficiency, three (5%) experienced cardiovascular complications, whereas only one (0.16%) patient with normal renal function developed such major complications.

**Table 3:** Frequencies and grading of perioperative 90-d major complications in patients who underwent radical cystectomy and pelvic lymph node dissection.

Major comlications	CDC grading	Normal renal function(n=24)	Preoperative renal insufficiency(n =10)
Gastrointestinal complications		8 (1.24%)	2 (3.33%)
Mechanical intestinal obstruction	III	4	1
Ileo-pouch fistual	III	1	0
Colon- pouch fistual	III	2	1
Enterocutaneous fistual	III	I	0
Cardic complications		1 (0.16%)	3 (5%)
Myocardial infarction	IV	0	2
Heart failure	IV	1	0
	V	0	1
Pulmonary complications		2 (0.31%)	1 (1.67%)
Respiratory failure	IV	1	1
Pneumothorax	III	1	0
Neurological complications		5 (0.78%)	2 (3.33%)
Acute cerebral infarction	IV	4	2
	V	1	0
Wound compliactions		8 (1.24%)	2 (3.33%)
Fascial dehiscence/ evisceration	III	8	2

CDC, Clavien-Dindo classification.

### 3.3 Propensity score matching

In order to reduce the selection bias, a one-to-one PSM was performed (**Table 4**). In total, 118 patients (59 with normal preoperative renal function and 59 with preoperative renal insufficiency) were successfully matched. After matching, the two groups had no significant difference in terms of age, ASA score, pathological T stage, and pathological N stage. A comparison of complications after matching was carried out for the sensitivity analysis (**Table 5**). After matching, the patients in the preoperative renal insufficiency group had higher CDCs and a higher incidence of major complications (16.9% vs 1.7%, *p* = 0.004).

**Table 4:** The clinicopathologic characteristics of the patients before matched and after matched.

	before mathed			after matched		
	Normal renal function (n=645)	Preoperative renal insufficiency (n=60)	*p*	Normal renal function (n=59)	Preoperative renal insufficiency (n=59)	*p*
Age	67 (58-73)	69 (60-76)	0.067	70 (60-76)	70 (61-77)	0.885
ASA score			<0.001			0.848
≤2	552 (85.6%)	38(63.3%)		37 (62.7%)	38(64.4%)	
>2	93(14.4%)	22(36.7)		22(37.3%)	21(35.6)	
Type of urinary diversion			0.014			0.022
Ureterocutaneostomy	166 (25.7%)	26 (43.3%)		12 (20.3%)	25 (42.4%)	
Ileal conduit	447 (69.3%)	33 (55.0%)		46 (78%)	33 (55.9%)	
Orthotopic neobladder	32 (5%)	1 (1.7%)		1 (1.7%)	1 (1.7%)	
Pathological T stage			<0.001			0.533
Ta and Tis and T1	189 (29.3%)	5 (8.3%)		9 (15.3%)	5 (8.5%)	
T2	204 (31.6%)	15 (25.0%)		10 (16.9%)	15 (25.4%)	
T3	195(30.2%)	30 (50.0%)		30(50.8%)	30(50.8%)	
T4	57(8.8%)	10 (16.7%)		10(16.9%)	9 (15.3%)	
Pathologic nodal stage			0.027			0.499
N0	553(85.7%)	45(75%)		48(81.4%)	45(76.3%)	
N+	92(14.3%)	15(25%)		11(18.6%)	14(23.7%)	

ASA, American Society of Anesthesiologists.

**Table 5:** Comparasion of complications for patients after matching.

	Normal renal function (n = 59)	Preoperative renal insufficiency (n = 59)	*p*
Clavien-Dindo class			<0.001
0	3 (5.1%)	14 (23.7%)	
1	0	0	
2	55 (93.2%)	35 (59.53%)	
3	0	4 (6.8%)	
4	1 (1.7%)	5 (8.5%)	
5	0	1 (1.7%)	
Major complication	1 (1.7%)	10 (16.9%)	0.004

## 4 Discussion

In our analysis of the radical cystectomy database, we investigated the correlation between preoperative renal insufficiency and the occurrence of major complications following a radical cystectomy for bladder cancer. The study yielded three principal findings. First, patients with renal insufficiency exhibited a higher CDC grade for postoperative complications, with a particularly elevated incidence of major complications. Second, there was a significant positive correlation between preoperative renal function and the incidence of major complications. Third, preoperative renal insufficiency was identified as a risk factor for the development of major complications.

A radical cystectomy has a high morbidity due to its difficulty and surgical trauma. According to the data of previous studies, the incidence of postoperative complications ranges from 31.5% to 64% and 13% to 35% of patients suffer from more than one major complication after a radical cystectomy (3–11). In this study, the incidence of 90-day complication rates was 71%, which was higher than the data reported in the literature. This variation of postoperative complications was caused by the interobserver variability in defining and grading complications, and, through a rigorous evaluation of clinical data, a greater number of complications would be detected, especially grade 1 and 2 complications. Furthermore, the 90-day major complication rate was 4.8% in our center, which was lower than the data reported (23). This could be due to an improvement in perioperative management in our institution.

Different surgical access methods can influence the incidence of postoperative complications. Existing literature has confirmed that a LRC was associated with a lower rate of postoperative complications compared to a ORC (24). However, there were no statistically significant differences in surgical access methods between the patients with normal preoperative renal function and those with preoperative renal insufficiency (**Table 1**). We conducted subgroup analyses to compare the incidence of postoperative complications between the patients with normal renal function and those with preoperative renal insufficiency across different surgical access methods (**Supplementary Table 1**). For patients who underwent a ORC, the incidences of postoperative complications (*p* = 0.002) and major complications (19.5% vs. 4.4%, *p* = 0.001) were significantly higher in the preoperative renal insufficiency group. In contrast, for patients who underwent a LRC, the incidence of major postoperative complications was higher in the preoperative renal insufficiency group (10.5% vs. 2.1%, *p* = 0.091), although this difference was not statistically significant, which may be attributed to the small sample size. Therefore, the surgical access method may not mitigate the increased risk of postoperative complications associated with preoperative renal insufficiency.

We used serum creatinine levels instead of glomerular filtration rate to assess renal function primarily because serum creatinine testing is convenient and the clinical data was readily available. Furthermore, although creatinine levels are influenced by various factors, studies have shown that serum creatinine correlates well with glomerular filtration rate, particularly in patients with renal impairment (25). A large proportion of the patients who undergo a radical cystectomy have renal dysfunction. Some of these patients have a history of chronic kidney disease, while most of them have an invasion of the distal ureter due to an advanced tumor, leading to postrenal obstruction (26). In our cohort, among the patients with renal insufficiency, 38 (63.3%) patients had an invasion of the distal ureter, and these patients all had posterior renal insufficiency. From the results given in **Table 1**, patients with preoperative renal insufficiency had higher pathological T stages (*p* < 0.001), and higher pathological nodal stages (25% vs 14.3%, *p* = 0.027), which indicated that patients with preoperative renal insufficiency group had more advanced tumors.

Previous studies indicated that patients with preoperative renal insufficiency who undergo a radical cystectomy were at a higher risk of renal function deterioration (27), and preoperative renal insufficiency was associated with worse oncological outcomes postoperatively (28, 29). In this study, we found that patients with preoperative renal insufficiency had a higher incidence of major complications, and preoperative renal insufficiency was a risk factor for major complications in patients after a radical cystectomy. This finding was consistent with the results of our previous study, although this was analyzed in a different population (17). In addition, preoperative renal insufficiency was associated with major complications for elderly patients after a radical cystectomy, when the endpoint was a comprehensive complication index (CCI) ≥33.7 (18). Wuethrich et al. reviewed the predictors for 90-day postoperative major complications and also found preoperative renal insufficiency to be an independent predictor (11). Not only for radical cystectomy, a previous study identified renal dysfunction to be an important risk factor for death after non-cardiac surgery, and the risk increases for patients with moderate to severe kidney dysfunction (30). We also compared the major complications between the two groups and the incidence of cardiac complication in the preoperative renal insufficiency group was higher than in the normal renal function group. This result indicated that the presence of impaired kidney function before a radical cystectomy may be associated with an increased risk of cardiac complications, which was proved in the previous study on non-cardiac surgery (31).

Effectively reducing postoperative complications can be achieved through enhanced perioperative management, such as the application of enhanced recovery after surgery (ERAS) protocols. Studies have confirmed that ERAS contributes to improve complication rates, decrease hospital length-of-stay, and/or time to bowel recovery (32–35). Additionally, identifying and correcting patient risk factors preoperatively are also effective (23, 36). The etiology of renal insufficiency can be categorized into pre-renal, renal, and post-renal causes. In the context of bladder cancer patients, the majority of renal impairments are attributed to post-renal causes, specifically due to the invasion of the ureteral orifices by advanced-stage tumors. Preoperative evaluation of the cause of preoperative renal insufficiency is necessary. For patients with preoperative post-renal renal insufficiency, a percutaneous nephrostomy is a viable method to ameliorate renal function prior to the surgery. The restoration of renal function may mitigate the risk of major complications. Furthermore, improved renal function could potentially enhance the tolerability of neoadjuvant chemotherapy, a treatment modality that has been shown to offer oncological benefits in terms of downstaging and improving survival outcomes (37).

With the advancement of minimally invasive techniques, robot-assisted radical cystectomy (RARC) has been increasingly adopted in various medical centers. According to the literature, the incidence of postoperative complications following RARC ranges from 62% to 67% (15, 38, 39). RARC offers comparable oncological control to open radical cystectomy while reducing the incidence of complications and shortening hospital stays (12, 15). Consequently, there is a need for further clinical research to explore the relationship between preoperative renal insufficiency and the incidence of postoperative complications in patients undergoing RARC for bladder cancer.

This study has some limitations. First, its retrospective nature introduces inherent risks of selection bias. To mitigate this, we employed a multivariate regression analysis, which allowed us to control for potential confounding factors as rigorously as possible. Second, the absence of a large cohort of patients with preoperative renal insufficiency who underwent a nephrostomy to ameliorate renal function prior to a radical cystectomy is a significant constraint. This limitation affects the generalizability of our findings. Therefore, we advocate for future multi-institutional studies to provide external validation of our results. Third, intraoperative and long-term complications were not described because of a lack of data. It would be interesting to compare intraoperative complications and postoperative complications at different terms in a further study.

## 5 Conclusions

In our cohort, patients with preoperative renal insufficiency exhibited a higher incidence of complications following a radical cystectomy and renal insufficiency was a significant risk factor for major complications.

## Data Availability

The raw data supporting the conclusions of this article will be made available by the authors, without undue reservation.
